# Wearing face masks in public during the influenza season may reflect other positive hygiene practices in Japan

**DOI:** 10.1186/1471-2458-12-1065

**Published:** 2012-12-10

**Authors:** Koji Wada, Kuniko Oka-Ezoe, Derek R Smith

**Affiliations:** 1 Department of Public Health, Kitasato University School of Medicine, 1-15-1 Kitasato, Minami-ku, Sagamihara, Kanagawa, 252-0374, Japan; 2 School of Health Sciences, Faculty of Health, University of Newcastle, Brush Road, Ourimbah, New South Wales, 2258, Australia

**Keywords:** Face mask, Health behavior, Hygiene practices, Influenza

## Abstract

**Background:**

Although the wearing of face masks in public has not been recommended for preventing influenza, these devices are often worn in many Asian countries during the influenza season. In Japan, it is thought that such behavior may be an indicator of other positive hygiene practices. The aim of this study, therefore, was to determine if wearing a face mask in public is associated with other positive hygiene practices and health behaviors among Japanese adults.

**Methods:**

We initially recruited around 3,000 Japanese individuals ranging from 20 to 69 years of age who were registered with a web survey company. Participants were asked to recall their personal hygiene practices during the influenza season of the previous year. Logistic regression analysis was then used to examine the associations between wearing a face mask in public and personal hygiene practices and health behaviors.

**Results:**

A total of 3,129 persons responded to the survey, among whom 38% reported that they had worn a face mask in public during the previous influenza season. Wearing a face mask in public was associated with various self-reported hygiene practices including: frequent hand washing (adjusted Odds Ratio [OR]: 1.67; 95% Confidence Interval [95%CI]: 1.34-1.96), occasional hand washing (OR: 1.43; 95%CI: 1.10-1.75), frequently avoiding crowds (OR: 1.85; 95%CI: 1.70-1.98), occasionally avoiding crowds (OR: 1.65; 95%CI: 1.53-1.76), frequent gargling (OR: 1.68; 95%CI: 1.51-1.84), occasional gargling (OR: 1.46; 95%CI: 1.29-1.62), regularly avoiding close contact with an infected person (OR: 1.50; 95%CI: 1.33-1.67), occasionally avoiding close contact with an infected person (OR: 1.31; 95%CI: 1.16-1.46), and being vaccinated of influenza in the last season (OR: 1.31; 95%CI: 1.17-1.45).

**Conclusions:**

Overall, this study suggests that wearing a face mask in public may be associated with other personal hygiene practices and health behaviors among Japanese adults. Rather than preventing influenza itself, face mask use might instead be a marker of additional, positive hygiene practices and other favorable health behaviors in the same individuals.

## Background

Wearing a face mask in public has not been recommended for preventing influenza virus infection [1]. Even though a face mask might provide some protection from inhaling larger droplets and hindering hand contact to the mouth and nose, the mask itself does not fit tightly enough to block droplets from entering between the face and mask [2]. In some Asian countries including Japan, many healthy people wear face masks in public during the influenza season, with an expectation that it helps prevent respiratory infections [3,4].

The effectiveness of wearing a face mask in certain settings with a high risk of influenza infection such as staying with influenza patients at home and shared living setting has been demonstrated in some studies. When a face mask is used correctly by infected individuals, it may help prevent household transmission by hindering the spread of infective respiratory droplets [5-7]. Wearing a facemask as well as adopting positive hand hygiene practices has been shown to reduce respiratory illness in shared living settings among young adults [8,9] with a relatively high compliance when wearing a mask. However, some other research has shown that compliance when wearing a face mask is low, especially in non-randomized studies [10,11].

On the other hand, it is reasonable to suggest that wearing face masks in the community might be an indicator of positive personal hygiene practices, health behaviors and perceptions of disease prevention. Despite this fact, no studies appear to have explored the association between face mask use and other personal hygiene practices and health behaviors. Aside from the potential public health benefits, examining these associations would also be very helpful to help minimize statistical confounding during research on the effectiveness of face masks in non-randomized studies. The aim of this study, therefore, was to determine if wearing a face mask in public is associated with other hygiene practices and health behaviors among Japanese adults.

## Methods

### Data collection

This study originally sought to recruit 3,000 Japanese individuals aged 20 to 69 years who were registered in a web survey company (among randomly selected 7,937 persons in a total 1.60 million registrants) in September 2011. The web survey company invited adults who were interested in being part of a survey that included some financial incentives for participating. The company then requested selected registrants to respond and ceased recruitment when the total number of participants exceeded our target of 3,000 individuals. Participants were classified into 5 groups by age range: 20–29, 30–39, 40–49, 50–59 and 60–69 years, as we estimated that a sample size of 300 participants per age group for each gender would be required. Individuals who agreed to participate in the study then completed an anonymous online questionnaire which included various questions regarding their hygiene practices and health behaviours during the October 2010 to March 2011 influenza season in Japan.

Questions included basic demographic information (age and sex), hygiene practices (wearing a face mask in public, hand washing, gargling, and avoiding crowds and infected people) and health behaviours (sleep quality and influenza vaccination status). We also asked the following questions about hygiene practices: “As a method to prevent influenza, to what extent do you practice the following methods? For each question, select the implementation status that applies to you the most during the periods when influenza spreads (winter).” For this section, we provided six items, as follows: “I wear a surgical mask in public during influenza season,” “I wash my hands,” “I try to avoid going to places where there are lots of people,” “I gargle,” “I try not to go near people who are infected,” and “I try to have a good sleep.” For each item, a four-point scale was provided, with the options: “I do this frequently,” “I do this occasionally,” “I don’t do this often,” and “I don’t do this.” We also asked the participants to indicate their influenza vaccination status in the previous winter.

### Statistical analysis

Logistic regression analysis was used to explore statistical associations between wearing a face mask in public during the influenza season and personal hygiene practices and health behaviors. Results were displayed as Odds Ratios (OR) with 95% Confidence Intervals (95% CI). We combined the responses: “I don’t do this often” and “I don’t do this” as the reference group. We also combined: “do frequently” and “do occasionally” as outcomes for the question: “I wear a surgical mask in public during influenza epidemic season.” We initially examined the variables using univariate analysis, and then by multivariate analysis to include factors that had been shown to be significant at the P=0.10 level during univariate analysis. All analyses were performed using IBM SPSS Statistics 19. As the outcome of interest (the incidence of household transmission) was common and this can affect the approximation of relative risk [12], we used Zhang’s formula to correct the odds ratios [13].

### Ethics

This study was approved by the Human Research Committee of the Kitasato University School of Medicine in Japan.

## Results

A total of 3,129 individuals, including 1,549 males and 1,580 females, participated in the study (Table 1). Approximately 20% were distributed in each age group. Participants who answered that they frequently or occasionally wore a face mask in public constituted 38.4% of the total participants (n=1,203). Table 2 indicates personal hygiene practices and other health behaviors by face mask wearing status. The highest proportion of face mask wearing was reported by the oldest age group (60–69 years), while the lowest proportion was reported by the youngest age group (20–29 years). Approximately half of all female participants reported wearing a face mask, while only a third of all male participants reported having done so. High proportions of both the face mask and non-face mask wearing groups reported hand washing. Compared to the non-face mask wearing group, a larger proportion of the face mask wearing group reported that they undertook regular gargling.

**Table 1:** Participants characteristics and responses

	**n=3129**	**(%)**
Age (years)		
20–29	618	(19.8)
30–39	628	(20.1)
40–49	627	(20.0)
50–59	632	(20.2)
60–69	624	(19.9)
Sex		
Male	1549	(49.5)
Female	1580	(50.5)
I wear a face mask in public during the influenza season		
Frequently	476	(15.2)
Occasionally	727	(23.2)
Not often/not do	1926	(61.6)
I wash my hands		
Frequently	1799	(57.5)
Occasionally	1098	(35.1)
Not often/not do	232	(7.4)
I try to avoid going to places where there are lots of people		
Frequently	504	(16.1)
Occasionally	1318	(42.1)
Not often/not do	1307	(41.8)
I gargle		
Frequently	1230	(39.3)
Occasionally	1110	(35.5)
Not often/not do	789	(25.2)
I avoid close contact with people who are infected		
Frequently	769	(24.6)
Occasionally	1621	(51.8)
Not often/not do	739	(23.6)
I try to have a good sleep		
Frequently	971	(31.0)
Occasionally	1512	(48.3)
Not often/not do	646	(20.6)
I was vaccinated last influenza season		
Yes	500	(16.0)
No	2629	(84.0)

**Table 2:** Personal hygiene practices and other health behaviors by face mask wearing status

	**Wearing a face mask**		**Not wearing a face mask**	
	**n =1203**	**(%)**	**n = 1926**	**(%)**
Age (years)				
20–29	194	(31.4)	424	(68.6)
30–39	246	(39.2)	382	(60.8)
40–49	249	(39.7)	378	(60.3)
50–59	242	(38.3)	390	(61.7)
60–69	272	(43.6)	352	(56.4)
Sex				
Male	467	(30.1)	1082	(69.9)
Female	736	(46.6)	844	(53.4)
I wash my hands				
Frequently	890	(49.5)	909	(50.5)
Occasionally	293	(26.7)	805	(73.3)
Not often/not do	20	(8.6)	212	(91.4)
I try to avoid going to places where there are lots of people				
Frequently	321	(63.7)	183	(36.3)
Occasionally	626	(47.5)	692	(52.5)
Not often/not do	256	(19.6)	1051	(80.4)
I gargle				
Frequently	688	(55.9)	542	(44.1)
Occasionally	394	(35.5)	716	(64.5)
Not often/not do	121	(15.3)	668	(84.7)
I avoid close contact with people who are infected				
Frequently	440	(57.2)	329	(42.8)
Occasionally	625	(38.6)	996	(61.4)
Not often/not do	138	(18.7)	601	(81.3)
I try to have a good sleep				
Frequently	493	(50.3)	488	(49.7)
Occasionally	563	(37.0)	959	(63.0)
Not often/not do	147	(23.5)	479	(76.5)
I was vaccinated last influenza season				
Yes	228	(43.8)	292	(56.2)
No	975	(36.8)	1674	(63.2)

Logistic regression analysis revealed that wearing a face mask in public and personal hygiene practices and health behaviors were significantly associated (Table 3). Wearing a face mask in public was associated with various self-reported hygiene practices including: frequent hand washing (adjusted Odds Ratio [OR]: 1.67; 95% Confidence Interval [95%CI]: 1.34-1.96), occasional hand washing (OR: 1.43; 95%CI: 1.10-1.75), frequently avoiding crowds (OR: 1.85; 95%CI: 1.70-1.98), occasionally avoiding crowds (OR: 1.65; 95%CI: 1.53-1.76), frequent gargling (OR: 1.68; 95%CI: 1.51-1.84), occasional gargling (OR: 1.46; 95%CI: 1.29-1.62), regularly avoiding close contact with an infected person (OR: 1.50; 95%CI: 1.33-1.67), occasionally avoiding close contact with an infected person (OR: 1.31; 95%CI: 1.16-1.46), regularly trying to have good sleep (OR:1.17; 95%CI: 1.01-1.34), occasionally trying to have good sleep (OR:1.19; 95%CI: 1.04-1.34) and being vaccinated against influenza (OR: 1.31; 95%CI: 1.17–1.45).

**Table 3:** Statistical associations between wearing a face mask in public and personal hygiene practices and health behaviors

**Variables**	**Crude**		**Adjusted**	
	**OR**	**(95% CI)**	**OR**	**(95% CI)**
I wash my hands				
Frequently	2.25	(2.09–2.37)	1.67	(1.34–1.96)
Occasionally	1.84	(1.56–2.07)	1.43	(1.10–1.75)
I try to avoid going to places where there are lots of people				
Frequently	2.13	(2.04–2.21)	1.85	(1.70–1.98)
Occasionally	1.82	(1.72–1.91)	1.65	(1.53–1.76)
Not often/not do	1		1	
I gargle				
Frequently	2.12	(2.02–2.20)	1.68	(1.51–1.84)
Occasionally	1.70	(1.56–1.83)	1.46	(1.29–1.62)
Not often/not do	1		1	
I avoid close contact with people who are infected				
Frequently	2.04	(1.93–2.14)	1.50	(1.33–1.67)
Occasionally	1.64	(1.51–1.76)	1.31	(1.16–1.46)
Not often/not do	1		1	
I try to have a good sleep				
Frequently	1.75	(1.62–1.87)	1.17	(1.01–1.34)
Occasionally	1.42	(1.28–1.55)	1.19	(1.04–1.34)
Not often/not do	1		1	
I was vaccinated last influenza season				
Yes	1.19	(1.06–1.31)	1.31	(1.17–1.45)
No	1		1	

OR: adjusted Odds ratio; CI: confidence interval.

## Discussion

This study provides some compelling evidence that wearing a face mask in public is associated with other positive personal hygiene practices and health behaviors among Japanese adults. Participants who wore a face mask were more likely to report practicing additional preventive hygiene measures including hand washing, gargling, avoiding crowds and close contact with ill people, having good quality sleep and being vaccinated against influenza.

Previous research conducted elsewhere has also elucidated the prevalence of hygiene practices with respect to influenza. A study from Korea for example, conducted during the early phases of the influenza pandemic in 2009, reported that 57% of female and 34% of male participants washed their hands five times a day [14]. Similarly, Lau and colleagues reported that 22% of their study subjects in Hong Kong wore face masks regularly in public during the early phases of the pandemic, while 45% of them washed their hands more than 10 times [15]. The prevalence of positive hygiene practices reported in studies conducted during the 2009 influenza pandemic appears to be higher than during the ‘general’ influenza season. In fact, a much lower prevalence of preventive measures than that of the 2009 pandemic have been observed, even in outbreak situations. For example, less than 10% of participants implemented preventive measures (including frequent hand washing, wearing face masks and getting more sleep) in a study of Dutch and Finnish individuals during the SARS outbreak of 2003 [16]. Presumably, the risk of being affected by a disease within a community and country influences the distribution and uptake of preventive hygiene measures.

We hypothesize that various factors probably contributed to the significant associations between face mask use and other hygiene practices and health behaviors during the Japanese influenza season. Firstly, they might reflect a high level of social and cultural acceptance of hygiene practices and health behaviors in this country [4], generally, as personal preventive measures are deeply engrained in cultural attitudes and behaviors within the Japanese community – including in the workplace [17]. All hygiene practices and health behaviors investigated during the current study are recommended by Japanese health authorities such as the Ministry of Health, Labour, and Welfare. Secondly, it is reasonable to suspect that individual risk perceptions might have influenced the statistical association we elucidated in the current study. An investigation from Hong Kong, for example, looked at hygiene behaviors during the early phases of the influenza A(H1N1) 2009 pandemic [15] and revealed that wearing a face mask in public was associated with a perception of the effectiveness of face mask use versus the dangers of influenza A(H1N1). Although previous studies have been conducted in culturally different settings and at different times (such as in the early phase of a pandemic), the current research nevertheless suggests that significant associations might have been influenced by individual perceptions. As such, further studies regarding the impact of individual perceptions are needed for verification [18].

Although the effectiveness of wearing a face mask for preventing infectious diseases has been investigated in various other studies [5,7,9,10,19,20], most have not considered possible associations between wearing a face mask and additional hygiene practices. However, we should note that a randomized controlled study which allocated face masks only revealed no statistically significantly differences in hand hygiene practices [8]. As such, it can be seen that any additional research to assess the contribution of face masks in preventing respiratory infections, will clearly need to monitor other health behaviors as part of their investigation.

Given that our research was one of the first of its kind, we acknowledge that the study might have incurred some limitations. Firstly, the generalizability of the results might be limited because the study participants were recruited using an online survey tool. This population would, presumably, have had internet access and therefore, might be more aware of preventive measures against influenza – especially those promoted on the internet [21]. In addition, as this study utilized an internet survey, we do not have any information on the non-respondents. Secondly, there is the possibility that it may be difficult for people to accurately recall their hygiene practices of the previous year in detail. A bias may exist among people who wear masks if they are more inclined to report undertaking other positive hygiene practices at higher rates than individuals who report lower mask use. Thirdly, as our current study was conducted in only one country (Japan), further research is therefore needed to determine the situation in other countries, especially those with a relatively lower rate of face mask use in the general population. Lastly, given that our study was cross-sectional in design, we are unable to confirm the existence of causal relationships.

## Conclusions

Overall, this study suggests that wearing a face mask in public may be associated with other personal hygiene practices and health behaviors among Japanese adults. Rather than preventing influenza itself, face mask use might instead be a marker of additional, positive hygiene practices and other favorable health behaviors in the same individuals.

## Competing interests

The authors declare that they have no competing interests.

## Authors’ contribution

KW conceived and implemented the study. All the authors contributed to writing and revising the manuscript. All authors read and approved the final manuscript.

## Pre-publication history

The pre-publication history for this paper can be accessed here:

http://www.biomedcentral.com/1471-2458/12/1065/prepub
